# The Impact of Acute Nutritional Interventions on the Plasma Proteome

**DOI:** 10.1210/clinem/dgad031

**Published:** 2023-01-20

**Authors:** Spyros I Vernardis, Vadim Demichev, Oliver Lemke, Nana-Maria Grüning, Christoph Messner, Matt White, Maik Pietzner, Alina Peluso, Tinh-Hai Collet, Elana Henning, Christoph Gille, Archie Campbell, Caroline Hayward, David J Porteous, Riccardo E Marioni, Michael Mülleder, Aleksej Zelezniak, Nicholas J Wareham, Claudia Langenberg, I Sadaf Farooqi, Markus Ralser

**Affiliations:** Molecular Biology of Metabolism Laboratory, The Francis Crick Institute, London, NW1 1HT, UK; Department of Biochemistry, Charité—Universitätsmedizin Berlin, 10117 Berlin, Germany; Department of Biochemistry, Charité—Universitätsmedizin Berlin, 10117 Berlin, Germany; Department of Biochemistry, Charité—Universitätsmedizin Berlin, 10117 Berlin, Germany; Molecular Biology of Metabolism Laboratory, The Francis Crick Institute, London, NW1 1HT, UK; Molecular Biology of Metabolism Laboratory, The Francis Crick Institute, London, NW1 1HT, UK; MRC Epidemiology Unit, University of Cambridge, Cambridge, CB2 0SL, UK; Computational Medicine, Berlin Institute of Health at Charité—Universitätsmedizin Berlin, 10117 Berlin, Germany; Molecular Biology of Metabolism Laboratory, The Francis Crick Institute, London, NW1 1HT, UK; Metabolic Research Laboratories and National Institute for Health Research Cambridge Biomedical Research Centre, Wellcome-Medical Research Council Institute of Metabolic Science, Addenbrooke's Hospital, University of Cambridge, Cambridge, CB2 0QQ, UK; Service of Endocrinology, Diabetology, Nutrition and Therapeutic Education, Department of Medicine, Geneva University Hospitals, 1211 Geneva, Switzerland; Metabolic Research Laboratories and National Institute for Health Research Cambridge Biomedical Research Centre, Wellcome-Medical Research Council Institute of Metabolic Science, Addenbrooke's Hospital, University of Cambridge, Cambridge, CB2 0QQ, UK; Department of Biochemistry, Charité—Universitätsmedizin Berlin, 10117 Berlin, Germany; Centre for Genomic and Experimental Medicine, Institute of Genetics and Cancer, University of Edinburgh, Edinburgh, EH4 2XU, UK; MRC Human Genetics Unit, Institute of Genetics and Cancer, University of Edinburgh, Edinburgh EH4 2XU, UK; Centre for Genomic and Experimental Medicine, Institute of Genetics and Cancer, University of Edinburgh, Edinburgh, EH4 2XU, UK; Centre for Genomic and Experimental Medicine, Institute of Genetics and Cancer, University of Edinburgh, Edinburgh, EH4 2XU, UK; Core Facility High Throughput Mass Spectrometry, Charité—Universitätsmedizin Berlin, 10117 Berlin, Germany; Molecular Biology of Metabolism Laboratory, The Francis Crick Institute, London, NW1 1HT, UK; Department of Biology and Biological Engineering, Chalmers University of Technology, SE-412 96, Gothenburg, Sweden; Institute of Biotechnology, Life Sciences Center, Vilnius University, Vilnius SE-412 96, Lithuania; Randall Centre for Cell & Molecular Biophysics, King's College London, New Hunt's House, Guy's Campus, SE1 1UL London, UK; MRC Epidemiology Unit, University of Cambridge, Cambridge, CB2 0SL, UK; MRC Epidemiology Unit, University of Cambridge, Cambridge, CB2 0SL, UK; Computational Medicine, Berlin Institute of Health at Charité—Universitätsmedizin Berlin, 10117 Berlin, Germany; Precision Healthcare University Research Institute, Queen Mary University of London, London, E1 1HH, UK; Metabolic Research Laboratories and National Institute for Health Research Cambridge Biomedical Research Centre, Wellcome-Medical Research Council Institute of Metabolic Science, Addenbrooke's Hospital, University of Cambridge, Cambridge, CB2 0QQ, UK; Molecular Biology of Metabolism Laboratory, The Francis Crick Institute, London, NW1 1HT, UK; Department of Biochemistry, Charité—Universitätsmedizin Berlin, 10117 Berlin, Germany

**Keywords:** caloric restriction, oral glucose tolerance test, plasma proteomics, APOC1, type 2 diabetes

## Abstract

**Context:**

Humans respond profoundly to changes in diet, while nutrition and environment have a great impact on population health. It is therefore important to deeply characterize the human nutritional responses.

**Objective:**

Endocrine parameters and the metabolome of human plasma are rapidly responding to acute nutritional interventions such as caloric restriction or a glucose challenge. It is less well understood whether the plasma proteome would be equally dynamic, and whether it could be a source of corresponding biomarkers.

**Methods:**

We used high-throughput mass spectrometry to determine changes in the plasma proteome of i) 10 healthy, young, male individuals in response to 2 days of acute caloric restriction followed by refeeding; ii) 200 individuals of the Ely epidemiological study before and after a glucose tolerance test at 4 time points (0, 30, 60, 120 minutes); and iii) 200 random individuals from the Generation Scotland study. We compared the proteomic changes detected with metabolome data and endocrine parameters.

**Results:**

Both caloric restriction and the glucose challenge substantially impacted the plasma proteome. Proteins responded across individuals or in an individual-specific manner. We identified nutrient-responsive plasma proteins that correlate with changes in the metabolome, as well as with endocrine parameters. In particular, our study highlights the role of apolipoprotein C1 (APOC1), a small, understudied apolipoprotein that was affected by caloric restriction and dominated the response to glucose consumption and differed in abundance between individuals with and without type 2 diabetes.

**Conclusion:**

Our study identifies APOC1 as a dominant nutritional responder in humans and highlights the interdependency of acute nutritional response proteins and the endocrine system.

The plasma proteome plays a central role in mediating the physiological response to changes in nutritional state and energy balance. Moreover, changes in the proteome may reveal biomarkers for metabolic diseases and for diseases that are influenced by metabolism. Recently, several new methods were developed for the profiling of the plasma proteome at scale, which can detect thousands of different proteins that span a huge concentration range of at least 9 orders of magnitude (1). However, many of these proteins are circulating proteins that leak from tissues and are transported to the liver or kidney for clearance. A much smaller number of proteins is known to directly function in the plasma. This physiologically active, highly abundant fraction of the plasma proteome contains at least 10 apolipoproteins (APOs) that bind and transport lipids (2–5). Other metabolism-critical plasma proteins include albumin (6); coagulation factors (7, 8); and proteins of the innate and adaptive immune system, including the complement cascade and acute-phase response (9).

Previous studies have shown that the plasma proteome responds to different dietary interventions (5, 10). For instance, 2 proteomic studies in obese individuals revealed a high number of plasma proteins within the apolipoprotein family and inflammatory proteins that respond to weight loss. Thus far, there is little information about how dynamically the plasma proteome adapts to changes in nutrition. However, both endocrine parameters and the metabolome adapt quickly to nutritional interventions such as acute caloric restriction (CR) (11), indicating the response could be rapid also at the proteome level.

We herein addressed the response of the plasma proteome to 2 complementary, short-term metabolic interventions: acute, time-limited caloric restriction during an experimental medicine study (CR study) (11); and an oral glucose tolerance test (OGTT) conducted as part of the Ely epidemiological study (12). Moreover, we validated the results by comparing the plasma proteome to those obtained for samples collected as part of another epidemiological investigation, Generation Scotland (GS) (13).

To be able to detect concentration changes in nutrient transport proteins, some of which are highly sequence homologous, we performed a data independent acquisition (DIA) mass spectrometric (MS) analysis of the neat plasma proteome (14). This technology provides highly precise measurements of the high-abundant plasma proteome fraction and facilitates the cost-effective processing of large sample series.

Our study shows that short-term dietary interventions can impact the plasma proteome substantially and change multiple protein abundances. The response includes well-known protein biomarkers of malnutrition, or proteins considered as biomarkers for other human disease (15, 16), but also includes proteins that have obtained no attention as metabolic biomarkers previously. Then, we compared the CR response detected in the proteome with that in the metabolome and examined associations with longer-term metabolic and endocrine parameters, metabolic risk factors, and medical indicators measured in 2 epidemiological cohort studies: Ely (12) and Generation Scotland (13).

## Methods

### Caloric Restriction Study Sample Collection

**Figure 1.**
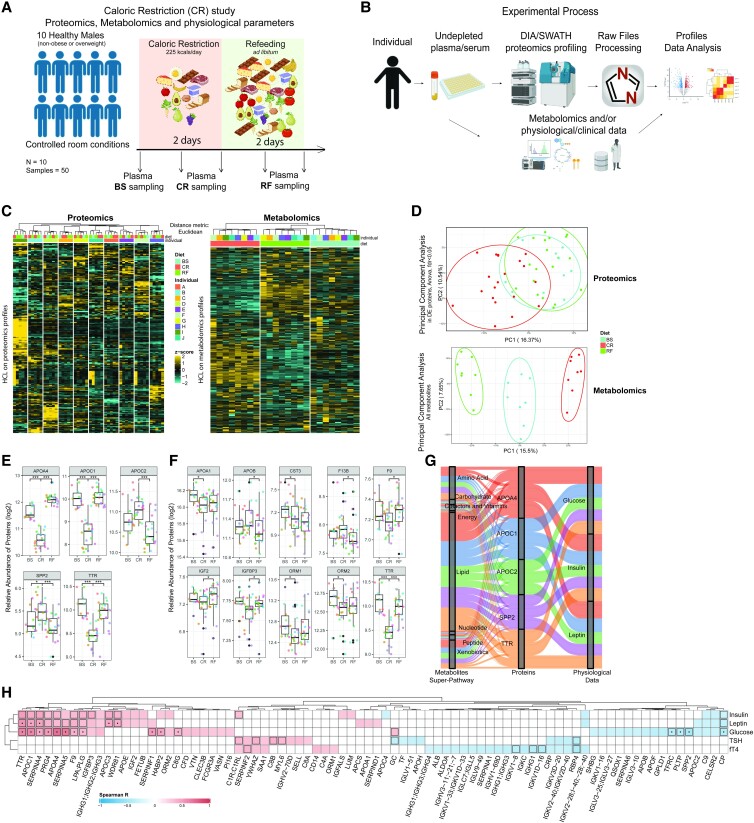
The proteomic response to an acute caloric restriction intervention. A) Design of the caloric restriction study. Ten healthy male individuals went under caloric restriction (CR, 2 days) and refeeding (RF, 2 days) in a clinical research facility (11). Plasma samples for proteomics were collected before the diet change (BS) and during CR and RF. Created with BioRender.com. B) Experimental methodology from sample collection to proteomic profiling by mass spectrometry (LC-SWATH-MS (14)) and data analysis (Methods). Created with BioRender.com. C) Hierarchical clustering (distance metric: Euclidean) of proteomic (left, 10 individuals) and metabolomic (right, 8 individuals) data expressed as heatmaps. D) Upper panel: principal component analysis (PCA) on differentially abundant proteins (ANOVA, FDR < 0.05) in the proteomic profiles over the CR experiment differentiate CR from BS and RF. Lower panel: PCA on metabolites indicates separation according to feeding state—BS, CR, and RF. E) Abundance changes in APOA4, APOC1, APOC2, SPP2, and TTR, proteins that represent the strongest response to acute CR in human plasma (*P < 0.05, ***P < 0.001). F) FDA-approved biomarkers differentially abundant in the CR and RF proteomes. Individuals show different responses based on diet in approved biomarkers. (*P < 0.05, ***P < 0.001). G) APOA4, APOC1, APOC2, SPP2, and TTR show association (Spearman, |cor| > 0.7) with metabolite concentration changes (metabolome) as classified at the super-pathway level and with blood glucose, insulin, and leptin levels, representing central endocrine and metabolic parameters. H) Correlation map of human plasma proteins with human physiological and endocrine parameters during the CR response (Spearman correlation, *P* < 0.05). Statistically significant correlations are colored; dots indicate statistical significance after row-wise multiple-testing correction (FDR < 0.05); black rectangles: column-wise. Glucose mmol/L, insulin pmol/L, leptin ng/mL, TSH mIU/L, fT4 pmol/L.

The study protocol and experimental design of the experimental medicine controlled human intervention study were described previously (11). The study was approved by the Cambridge local research ethics committee and was conducted in accordance with the principles of the Declaration of Helsinki. Written informed consent was received from each participant prior to inclusion in the study. We recruited 10 normal-weight healthy men using inclusion criteria as previously described (11). All males were healthy and not obese or overweight (average age: 23.8 years, average body mass index [BMI; kg/m^2^]: 23.3) (Supplementary Table S1 (17)). In the CR study, we only studied males, due to the known effects of leptin on the regulation of the hypothalamic–thyroid and gonadal axes, which may have confounded our analyses. Participants at baseline consumed a balanced diet (50% carbohydrate, 30% fat, and 20% protein). During caloric restriction, volunteers consumed 10% of normal energy requirement (226 kcal/d) for 2 days, again balanced (50% carbohydrate, 30% fat, and 20% protein), with the same macronutrient composition. After caloric restriction, volunteers were offered 3 substantial ad libitum buffet meals per day (20 MJ = 4777 kcal) and additional snacks (16 MJ = 3821 kcal) between meals for 2 days. They were invited to eat freely until comfortably full; food consumption was covertly measured. We collected fasting plasma samples at 8:00 Am at baseline, after CR and refeeding (RF) (Fig. 1A).

### Ely Study Design and Sample Collection

The Ely study, established in 1990, is a prospective study of the etiology of type 2 diabetes, as described in detail elsewhere (18, 19). The Ely study comprises individuals of European ancestry aged 50 to 79 years, registered at a single medical practice in Ely, Cambridgeshire, UK and evaluated in 3 phases. All participants of the Ely study gave their written informed consent, and the study was approved by the local ethics committee. A total of 200 participants (105 women/95 men) from Ely phase 3 with complete fasting and OGTT sample availability were selected for measurement (Supplementary Table S1 (17)). All participants attended a clinical examination for anthropometric measurements, medical questionnaires, and a 75-g OGTT. Glucose and insulin concentrations were measured at fasting and 30, 60, and 120 minutes after an oral glucose challenge. Proinsulin and 32-33 split proinsulin were measured at fasting. All measurements were taken on the OGTT day. None of the participants had a diagnosis of type 2 diabetes before the OGTT. After the OGTT, the cohort was grouped into individuals without diabetes (n = 128), those with impaired glucose tolerance (IGT) (n = 42), those with impaired fasting glucose (IFG) (n = 19), and people with diabetes (n = 10), according to the American Diabetes Association (20) criteria. Descriptive characteristics of phase 3 participants were previously published (21) and a summary of those is shown in Supplementary Table S1 (17).

### Generation Scotland Study Design and Sample Collection

Generation Scotland (GS) is a population-based cohort study of families from across Scotland (13). More than 24 000 participants (aged 18-99 years) were recruited between 2006 and 2011 when they completed questionnaires about health and lifestyle and donated blood samples for biomarker and -omics analyses after 4 hours of fasting. A subset of blood samples from 199 participants (average age: 56.51; average BMI: 26.34 kg/m^2^; sex distribution: 117 women/82 men) contributed to a pilot study and are considered here for the analyses (14). We note that the average BMI of the GS participants is higher than 25 kg/m^2^ (at 26.34 kg/m^2^) and hence the population sample is considered overweight, but not obese (BMI > 30 kg/m^2^). This BMI follows the overweight average of the UK (22).

All components of Generation Scotland received ethical approval from the NHS Tayside Committee on Medical Research Ethics (REC Reference Number: 05/S1401/89). All participants provided broad and enduring written informed consent for biomedical research. Generation Scotland has also been granted Research Tissue Bank status by the East of Scotland Research Ethics Service (REC Reference Number: 20/ES/0021), providing generic ethical approval for a wide range of uses within medical research. This study was performed in accordance with the Declaration of Helsinki.

Generation Scotland received core support from the Chief Scientist Office of the Scottish Government Health Directorates (CZD/16/6) and the Scottish Funding Council (HR03006) and is currently supported by the Wellcome Trust (216767/Z/19/Z). Genotyping of the GS:SFHS samples was carried out by the Genetics Core Laboratory at the Edinburgh Clinical Research Facility, University of Edinburgh, Scotland and was funded by the Medical Research Council UK and the Wellcome Trust (Wellcome Trust Strategic Award “STratifying Resilience And Depression Longitudinally” (STRADL) Reference 104036/Z/14/Z).

### Mass Spectrometry and Data Analysis for Proteomics

#### Materials

Mass spectrometric proteome analysis was performed using a recently described platform technology that combines a sample preparation workflow addressing the requirements for large serum and plasma cohort studies, analytical flowrate chromatography, and an optimized SWATH acquisition scheme (14). We used the following chemicals: urea (Honeywell Research Chemicals, 33247H), ammonium bicarbonate (Honeywell Research Chemicals, 40867), dithiothreitol (DTT, Sigma Aldrich, 43815), iodoacetamide (IAA, Sigma Aldrich, I1149), water (Alfa Aesar, 22934, ultrapure, HPLC Grade), trypsin (Promega, Sequence Grade, V5117), formic acid (FA, Thermo Fisher Scientific, 85178), acetonitrile (ACN, Fisher Chemicals, 10001334), C18 96-well plates (The Nest Group, BioPureSPE Macro 96-Well, 100 mg PROTO C18), and methanol (Fisher Chemicals, A456-212).

#### Protocol, plasma proteomics

To prepare the samples for proteome analysis, 5 μL of EDTA plasma sample were added into 50 μL 8 M urea, 0.1 M ammonium bicarbonate, pH 8.0, in order to denature proteins. Proteins were then reduced using 5 μL 50 mM dithiothreitol for 1 hour at 30 °C and alkylated with 5 μL 100 mM iodoacetamide for 30 minutes in the dark. The sample was diluted with 340 μL 0.1 M ammonium bicarbonate to 1.5 M urea. 200 μL of the solution was used for trypsinization. The proteins were digested overnight with trypsin (12.5 μL, 0.1 μg/μL) at 37 °C at a 1/40 trypsin/total protein ratio. Digestion was quenched with the addition of 25 μL 0.1% v/v FA. Peptides were cleaned up with C18 96-well plates, eluted with 50% v/v ACN, dried by a vacuum concentrator (Eppendorf Concentrator Plus, 5305000568), and redissolved in 50 μL 0.1% v/v FA to be processed by liquid chromatography (LC)-MS. Τhe methodology was extensively described in Messner et al (14).

The profiles for the CR cohort were acquired with 21-minute gradients using micro-flowrate chromatography, and the ones for the Ely and GS cohorts with 5-minute gradients using analytical flowrate chromatography. The GS study data were previously described (14). In more detail, for the CR study, the digested peptides were analyzed on a nanoAcquity (Waters) coupled to a TripleTOF 6600 (SCIEX). Then 2 μg of peptides were separated with a 23-minute nonlinear gradient starting with 3% B up to 40% B (Buffer A: 0.1% v/v FA; Buffer B: ACN/0.1% v/v FA) on a Waters HSS T3 column (150 mm × 300 µm, 1.8-µm particles) with a 5-µL/min flowrate. The DIA method consisted of an MS1 scan from m/z 400 to m/z 1250 (50 ms accumulation time) and 40 MS2 scans (35 ms accumulation time) with variable precursor isolation width covering the mass range from m/z 400 to m/z 1250. The samples were run in a random order. Quality control (QC) samples were run among every 10 sample runs.

For the Ely study, liquid chromatography was performed using the Agilent 1290 Infinity II system coupled to a TripleTOF 6600 mass spectrometer (SCIEX). A sample of 2 μg of peptides was separated using a C18 ZORBAX Rapid Resolution High Definition (RRHD) column (2.1 mm × 50 mm, 1.8-μm particles). A linear gradient was applied, ramping from 1% B to 40% B in 5 minutes (Buffer A: 0.1% v/v FA; Buffer B: ACN/0.1% v/v FA) with a flowrate of 600 μL/min. The mass spectrometer was operated in high-sensitivity mode. The DIA method consisted of an MS1 scan from m/z 100 to m/z 1500 (20 ms accumulation time) and 25 MS2 scans (25 ms accumulation time) with variable precursor isolation width covering the mass range from m/z 450 to m/z 850. Ion source gas 1 (nebulizer gas), ion source gas 2 (heater gas), and curtain gas were set to 30, 15, and 25 respectively. The source temperature was set to 450 °C and the ion spray voltage to 5500 V. Injections of samples took place in a random order.

Raw data were analyzed by DIA-NN with postprocessing analysis in R as described previously (14, 23). DIA-NN (version 1.7.12) was run in Robust LC (high precision) quantification mode, with maximum MS2 and MS1 mass tolerances set to 20 and 12 ppm, respectively. The scan window size was set to 6 for the Ely dataset and 11 for the CR dataset. Raw data processing was performed in 2 steps. First, the spectral library described previously was “refined” based on the respective SWATH dataset, with the original information in the library such as spectra and retention times being replaced by the empirical information obtained from the SWATH data, as enabled by the functionality of DIA-NN (24). Subsequently, the SWATH-based “refined” library (5) was used to re-analyze the respective dataset. During all steps, precursor false discovery rate (FDR) filtering was set to 1%. Postprocessing was carried out in R. The data were filtered at 1% gene group q-value. Only acquisitions with a minimum of 2000 precursor IDs were included. Intra-batch correction (14) based on repeat injections of QC samples was applied, using a linear method for the CR dataset and running median-based for the Ely dataset. The same QC samples were further used for inter-batch correction (14) for the Ely dataset. The quality of data acquisition is reflected by low coefficient of variation values and stability of the number of identified proteins, which were maintained in the 3 cohorts. The technical variation of the newly acquired proteome data was much lower compared to biological (Supplementary Fig. S1A (17)). Plasma proteomes were measured from the same samples as the metabolomes, so except the impact of storage (25–27), the datasets are fully matched.

### Metabolomics Analysis

Metabolomics data were collected as part of the CR study (11) and performed at Metabolon, Inc. (Durham, NC) with a methodology published before (28). Briefly, after methanol extraction, the samples were divided into fractions and our independent UPLC-MS/MS profiling methods were used: 2 reverse-phase LC with positive ion mode, focusing on hydrophobic or hydrophilic compounds, and 2 with negative ion mode. A Waters ACQUITY UPLC (Waters Corp., Milford, MA) and a Thermo Scientific Q-Exactive high resolution/accurate mass spectrometer (Thermo Fisher Scientific, Waltham, MA) at 35 000 mass resolution were used. The scan range was 70 to 1000 m/z and analytes were identified by automated comparison to a reference library of chemical standards.

### Statistical Analysis

Analysis of the acquired datasets was performed in the R programming platform (version 4.0.1). Only protein groups matching at least 2 different peptides were used in the final datasets. For differential abundance analysis, Wilcoxon test of the compare_means function with Benjamini-Hochberg correction of the *ggpubr* R package (29) was used. ANOVA was used for principal component analysis of differentially abundant proteins. Heatmaps and clusterings were done with the use of the *ComplexHeatmaps* R packages (30) in datasets with scaled values to the standard deviation, with Euclidean distance metric, after k-nearest neighbor imputation. Logarithmic values of the relative peak areas were used in most cases, as shown in the plots. The *ggplot2* R package (31) was used for the boxplots and scatter plot illustrations. For the PLS-DA the *mixOmics* R package was used (32). The *prcomp* R function was used for principal component analysis, with standard parameters. The *ggalluvial* R package (33) was used for the illustration of 3-part correlations among proteins, metabolites, and physiological (biochemical) measurements. The Spearman coefficient was used to describe correlations between proteins and physiological (biochemical) measurements in the CR, Ely, and GS studies.

The figures were built and saved in Adobe Illustrator v26.0.2 or with biorender.com.

## Results

### The Proteomic Response of the Plasma Proteome to Acute Caloric Restriction

Ten healthy, normal-weight male participants were housed in a clinical research facility (11). The study participants were exposed to acute CR by reducing the diet to 226 kcals ± 5 kcals/day (∼10% of the usual energy requirement) for 2 days, followed by an ad libitum refeeding (RF) period for the subsequent 2 days, during which diet, fluid intake, and timing of meals and sleep were monitored (11), Fig. 1A). Plasma samples for proteomics were collected and analyzed as part of this study (34) (PXD038526), while metabolomics and levels of plasma glucose, insulin, leptin, thyrotropin (thyroid-stimulating hormone), and free thyroxine (T4) were reported previously (11) (Supplementary Table S1 (17)). For each individual, 5 time points were determined: at the baseline (BS) point (after 24 hours of normal feeding), at day 1 and 2 of CR, and at day 3 and 4 of RF. In this sample set, mass spectrometry quantified 330 proteins, of which we consistently quantified 244 proteins in more than 90% of the samples (Supplementary Fig. S1 (17)).

Unsupervised hierarchical clustering based on Euclidean distance of the proteomes identified 10 discrete clusters that correspond to each individual (Fig. 1C). This result indicates that individual-specific proteomic signatures are maintained despite the acute CR condition. Moreover, the proteome captured interindividual differences in the actual CR response. Differentially abundant proteins included proteins of the adaptive and the innate immune systems, such as proteins of the complement cascade (eg, C1R, C4, C8A, C7, C9), coagulation factors (eg, F11, F2, F12), and multiple immunoglobulin regions (Supplementary Fig. S4 (17)). Indeed, an interaction between the immune system and nutritional responses was reported previously (35–37). Moreover, we detected interindividual differences in the response of metabolism-relevant proteins related to nutrient transport and fatty acid metabolism. This result indicated that interindividual responses to metabolic perturbation are depicted in the proteome (Supplementary Fig. S4 (17)).

Principal component analysis (PCA) (Fig. 1D) as well as a supervised analysis (partial least square-discriminant analysis [PLS-DA]) (Supplementary Fig. S2 (17)) separated baseline and refeeding from the CR response (Fig. 1D). Thirty-nine plasma proteins differed between baseline and acute CR (Wilcoxon test, FDR < 0.05, Supplementary Fig. S3 (17)). Five of the proteins responding to acute CR, apolipoproteins APOA4, APOC1, APOC2, secreted phosphoprotein 2 (SPP2), and transthyretin (TTR) showed, in combination, the highest relative response (largest median fold changes) and significance (lowest *P* values, Fig. 1E). Thirteen of the differentially concentrated proteins, many of which are nutrient transport proteins (APOB, APOC2, CELSR2, CFH, CLU, CPN1, F13B, GPDL1, PFN1, QSOX1, SPP2, TUBB1, VCL), were increased during CR and decreased upon RF, suggesting a high plasticity of the proteome when responding to nutritional changes. For instance, CR affected the concentration of serum amyloid P-component (APCS), an acute-phase protein that removes amyloids (38). Moreover, apolipoprotein C3 (APOC3) and cystatin C (CST3) levels decreased under CR but did not recover under RF phase. Inter-alpha-trypsin inhibitor heavy chain 3 (ITIH3), a protein correlated with obesity and diabetes (39), was the only protein that increased under CR and peaked under RF within the tested time frame.

### Apolipoproteins APOA4, APOC1, APOC2, SPP2, and TTR Dominate an Orchestrated CR Response at the Proteome and Metabolome Levels

We identified several proteins which changed in response to both CR and refeeding, including 10 proteins (60 biomarkers detected in total) that are known as disease biomarkers from previous studies (15, 16) (Fig. 1F). For instance, acute CR decreased cystatin C (CST3) levels. CST3 is known as a biomarker of kidney function since levels correlate with glomerular filtration rate (40). Its decrease in plasma during refeeding may suggest, among other factors (41), an increase in kidney function. Other examples include α1-acid glycoprotein ORM1 and α1-acid glycoprotein ORM2, acute-phase proteins that might regulate energy homeostasis (42). These proteins respond to CR and are restored under RF. Moreover, CR affected the levels of insulin-like growth factor 2 (IGF2), a growth factor important for fetal development as well as adipocyte proliferation (43). IGF2 decreased under CR and increased under RF. Insulin-like growth factor binding protein 3 (IGFBP3) has high homology to IGF2 and one of its roles is to facilitate IGF's transfer to receptors (44). Coagulation factors like F9 (factor IX) and F13B (factor XIIIB) are also among the proteins significantly affected by CR (Fig. 1F).

Next, we compared the proteome to metabolomic changes under acute CR that were determined for 8 of the study participants (11). Clustered in a similar fashion as the proteome, we obtained 3 discrete clusters of the metabolomic profiles that span over 1160 metabolite quantities of a broad range of metabolite classes. As previously reported ((11), “Methods”), the metabolome hence discreetly responds to the CR dietary intervention, ie, the baseline stage, the phases of CR, and the refeeding (BS, CR, RF), but no individual-specific clustering (like at the proteome level; Fig. 1C) was obtained. PCA (Fig. 1D) and supervised PLS-DA (Supplementary Fig. S2 (17)) confirmed this picture and fully separated BS, CR, and RF. In contrast to the proteome, where interindividual differences dominated the profiles, the metabolome mostly reflected the dietary intervention.

A correlation analysis (Spearman rank coefficients) of the proteomic vs the metabolomic data (Supplementary Fig. S5 (17)) highlighted 5 proteins (APOC1, APOA4, APOC2, TTR and SPP1; Fig. 1E) which correlated with metabolite concentration changes (Fig. 1G). The interactions of these proteins with the metabolome is consistent with their biochemical function. The apolipoprotein superclass is involved in lipid transport and metabolism (APOC1, APOA4, APOC2), while transthyretin (TTR) is a plasma transporter of thyroid hormones (45). The function of secreted phosphoprotein 2 (SPP2) is less well understood, but it could serve as a thiol protease inhibitor and could potentially increase inflammation (46) (Fig. 1G). APOC1, APOA4, and TTR correlated positively, while SPP2 levels correlated inversely with plasma glucose levels (Fig. 1G and 1H). Furthermore, APOC1 and TTR correlated with insulin and leptin levels, while APOC2 levels were inversely correlated with insulin levels (Fig. 1G and 1H). An overview of additional, significant correlations between CR-affected proteins and endocrine metabolic parameters indicates a broad interdependence of plasma proteome and physiology during the CR response (Fig. 1H). Many immunoglobulins, innate immune response proteins, complement proteins, and serine protease inhibitors (SERPIN) family members are correlated with metabolic parameters. Hence, the plasma proteome captures a response of the human plasma proteome to CR in a general and in an individual-specific manner. The dominating CR-affected proteins predominantly are nutrient transport proteins, and consistent with their biochemical function, they correlate with plasma metabolite concentrations and endocrine parameters. Notably our list of the most responsive and metabolome-correlated proteins included also proteins like SPP2, whose function in the human nutritional response is thus far barely understood.

### APOC1 Levels Change During an Oral Glucose Tolerance Test, and Differ Between People With Prediabetes and Type 2 Diabetes in the Ely Study

**Figure 2.**
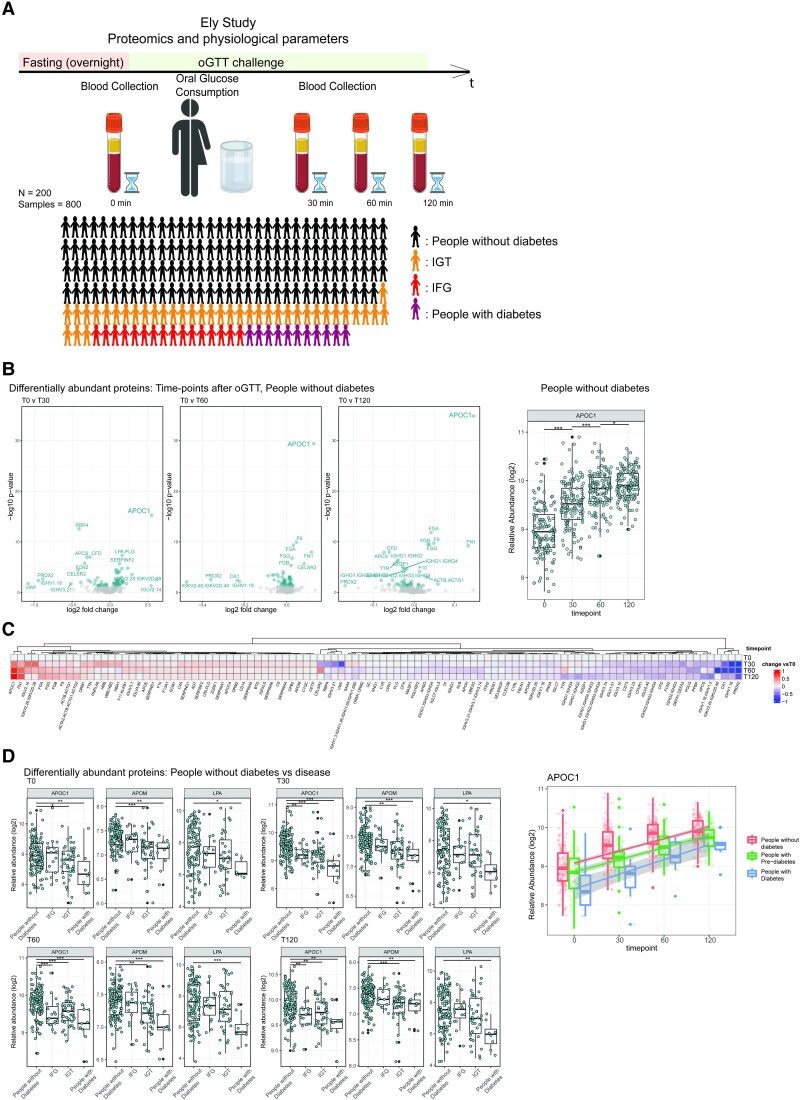
Proteome changes upon an oral glucose challenge highlight the role of APOC1 in nutritional responses. A) Oral glucose tolerance test (OGTT) in the Ely cohort (200 randomly chosen participants) and blood sample collection. Created with BioRender.com. B) Volcano plots comparing the proteome recorded at baseline (T0) with the proteomes record after 30, 60, and 120 minutes (T30, T60, T120) after glucose consumption. Proteins with *P* < 0.05 (Wilcoxon test) are highlighted. APOC1 abundance (boxplots) after OGTT in people without diabetes increases significantly at T30, T60, and T120 compared to T0. C) All differentially abundant proteins compared to T0 in people without diabetes. D) Differential abundance analysis between people with and without diabetes, IFG, and IGT individuals (Wilcoxon test) at time points 0, 30, 60, and 120 for apolipoproteins and lipoprotein-metabolism-related proteins reveals changes in APOC1, APOM, and LPA. (*P < 0.05, **P < 0.01, ***P < 0.001). E) APOC1 concentrations of people with and without diabetes, IGT, and IFG individuals after the OGTT challenge. Part of the figure was created with BioRender.com.

We next examined the acute response to positive energy balance, during an OGTT (12). The samples of 200 participants were collected after overnight fasting (T0), and 30, 60, or 120 minutes (T0 to T30, T60, and T120) after the oral administration of 75 g of glucose (47) (PXD039023) (Fig. 2A, “Methods”). None of the participants had a diagnosis of type 2 diabetes before the OGTT challenge (12, 18). After the test, the participants were categorized as those with diabetes or prediabetes (IFG, IGT) and those without, according to the criteria of the American Diabetes Association (20). Seventy-two of the participants met the criteria for diabetes or prediabetes (IFG, IGT). As more than 800 proteome samples had to be processed, we used a faster method based on analytical flowrate chromatography (14). The choice of method resulted in a high measurement precision but covered slightly fewer proteins (“Methods”). We detected 313 proteins, of which we consistently quantified 197 proteins in more than 90% of samples (Supplementary Fig. S1 (17), “Methods”).

We focused on 128 out of the 200 studied participants who, according to guidelines of the American Diabetes Association (20), were classified as people without diabetes. The proteomic analysis revealed multiple proteins that were increased or repressed by the glucose challenge (Wilcoxon test, *P* < 0.05) (Fig. 2B and 2C). The strongest quantitative response was detected in APOC1. The APOC1 levels were increased 1.46-fold at 30 minutes after the administration of the glucose, 1.73-fold after 60 minutes, and 1.86-fold after 120 minutes. (Fig. 2B). Other proteins affected by the OGTT were TTR, carbonic anhydrase (CA1), peroxiredoxin 2 (PRDX2), several actin molecules (ACTB, ACTG1, ACTA2), fibronectin (FN1), fibrinogens (FGA, FGB, FGG), and immunoglobulins (Fig. 2C).

We then compared the proteomes of individuals within the Ely cohort without diabetes (n = 128), to those with impaired glucose tolerance (IGTs, n = 42), with impaired fasting glucose (IFGs, n = 19), and with type 2 diabetes (n = 10). A subset of the plasma proteins, including APOM and LPA, were differentially abundant depending on these disease states. The most dominating response again was detected in APOC1 (Fig. 2D). APOC1 levels were higher in people without diabetes compared with people with diabetes (*P* < 0.01) and impaired glucose tolerance (*P* < 0.05) at T0 (Fig. 2D). Post-OGTT, people without diabetes reached higher APOC1 levels much faster (Fig. 2E) (*P* < 0.01, *P* < 0.001, *P* < 0.01 at T30, T60, T120, respectively).

We examined if the age of the Ely study participants may be a confounding factor. After grouping participants by decade of age (48–57, 58–70, 71–79), we observe a higher incidence of diabetes appear in the group aged 71 to 79 (Supplementary Fig. S10A (17)). Focusing on APOC1 and people without diabetes, we observe a small change in APOC1 abundance in the group aged 70 to 79 compared to the other 2 groups (*P* < 0.05) (Supplementary Fig. S10C (17)), and in younger groups, but only if individuals with diabetes are included in the analysis. Moreover, the signal is stronger in the 70 to 79 age group compared with the younger individuals. We hence speculate that an increased prevalence of diabetes with age results in lower APOC1 levels (Supplementary Fig. S10D (17)). Another potential confounder is sex differences. After a differential abundance analysis (Wilcoxon, *P* < 0.001) to interrogate the effects of sex in the Ely and GS studies, we observed differences in the abundance of some proteins, such as pregnancy zone protein (PZP) and sex hormone-binding globulin (SHBG) (Supplementary Fig. S11A and 11B (17)), proteins for which a sex-specific expression was reported previously (2, 59). Interestingly, we observe additional proteins such as GPLD1 (phosphatidylinositol-glycan-specific phospholipase D) and CP (ceruloplasmin) to be differentially abundant in a sex-specific manner—and particularly higher in men. Among the key markers of nutritional responses, only APOD shows a small but significant sex-specific trend of being more abundant in men.

### Changes in Acute Nutritional Response Proteins in Epidemiological Cohort Studies

**Figure 3.**
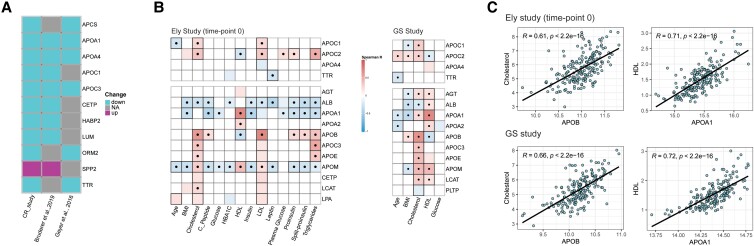
Proteins that dominate acute nutritional response correlate with metabolic parameters in 2 epidemiological studies: Ely and Generation Scotland. A) Proteins that are differentially abundant upon acute CR are also changed after 8 weeks of caloric restriction and weight loss in obesity, as determined by (5, 10). B) Correlation map (Spearman correlation, *P* < 0.05) of CR-responsive proteins, with metabolic and endocrine parameters recorded as part of the Ely and GS studies, respectively. Statistically significant correlations are colored; dots indicate statistical significance after column-wise multiple-testing correction (FDR < 0.05); black rectangles: row-wise. C) Example correlations of individual proteins with physiological measurements. HDL, Apolipoprotein (APOB, APOA1) levels correlate with cholesterol in Ely and GS studies (Spearman correlation). HDL mmol/L, cholesterol mmol/L.

Next, we asked if the responses detected are specific to the short nutritional interventions, or whether the affected pathways overlap with those affected by longer-term metabolic conditions. First, we compared our results with 2 recent proteomic studies that addressed caloric restriction for weight loss over an 8-week observational period in obese individuals (5, 10). We observed proteins changing after caloric restriction in all 3 studies (APOA1, APOA4, APOC3), or between our study and 1 of the other 2 (APCS, APOC1, CETP, HABP2, LUM, ORM2, SPP2, TTR) (Fig. 3A). These proteins changed in the same direction as in the short-term interventions. SPP2 was the only protein with an increasing abundance after caloric restriction, while increasing in the long-term condition of weight loss. Second, we compared our proteomic analysis to physiological data collected as part of the epidemiological studies, ie, the 200 individuals of the Ely study who participated in the OGTT test (12), and 200 individuals for the Generation Scotland cohort (13), for whom we have presented proteomes recently using the same technology (13) (Supplementary Table S1 (17)). We linked the proteome data to physiological parameters, including BMI, plasma glucose, venous whole-blood glucose, insulin, high-density lipoproteins (HDL), low-density lipoproteins (LDL), cholesterol, triglycerides, leptin, and glycated hemoglobin (HbA1c) (Fig. 3B). Most proteins that dominated the acute nutritional responses, and that were detected to respond to fasting and weight loss (5, 10), were correlated to endocrine parameters as determined as part of both studies. For instance, APOC1, APOC2, APOA4, and TTR were all associated with metabolic parameters (Spearman, FDR < 0.05) recorded as part of the Ely study, including BMI, cholesterol, leptin levels, plasma glucose levels, insulin and its precursors, triglycerides, or age (Fig. 3B, upper panel). Consistently with the function of apolipoproteins in lipoprotein complexes (3, 60), the levels of these apolipoproteins were also correlated with LDL and HDL values. Other physiological markers such as glycated hemoglobin (HbA1c) had a significant correlation with other protein members of the apolipoprotein superfamily (Fig. 3B, lower panel).

The proteome determined for 200 individuals randomly chosen from the Generation Scotland epidemiological study (GS study) confirmed this picture. GS is a family-based cohort and represents a population average (13). We previously had measured the proteome in serum samples obtained after 4 hours fasting (14). The analysis substantiated the picture provided by the Ely study. APOC1, APOC2, APOA4, and TTR, which are proteins that change in response to both short-term interventions, each correlated with at least one of the metabolic health parameters determined at the epidemiological level (Fig. 3B, upper panel). The proteomic data were linked to age, BMI, cholesterol, HDL, and glucose (13). For instance, APOM positively correlates with cholesterol in both GS and Ely studies. Reassuringly, some of the strongest correlations of proteins measured in both Ely and GS studies, with the endocrine parameters, were observed for proteins that have a functional relationship to the metabolome. For instance, cholesterol concentrations correlated with APOB levels, or lab values for LDL and HDL correlated with the concentration of their components APOB and APOA1, respectively (Fig. 3C). Indeed, APOB is part of triglyceride-rich lipoproteins, and APOA1 is a constituent of HDL and a marker for cardiovascular and metabolic disease (48, 49) (Fig. 3C).

## Discussion

The plasma plays an important role in mediating the systemic response to changes in nutritional status (5, 10). Recent proteomic technologies, such as the platform based on high-flowrate liquid chromatography and SWATH-MS applied here (14, 50), allow the measurement of large numbers of proteomes at high precision and low costs. Moreover, these technologies are attractive because they capture the physiologically active, highly abundant fraction of the plasma proteome that is involved, among other functions, in metabolite transport. Finally, because this plasma proteome fraction is analytically accessible, it is attractive for the rapid translation of marker panels into clinical use, as shown, for instance, during the COVID-19 pandemic (51).

We measured the plasma proteome of the acute human response to 2 short-term metabolic interventions, one inducing a negative energy balance (CR) followed by recovery (RF), and one with a positive energy balance as induced by an oral glucose challenge (OGTT) (34) (PXD038526). We found that short-term nutritional interventions result in a substantial change in the proteome. For instance, the CR experiment affected more than a tenth of the high-abundant functional plasma proteins in all study participants. Our data also reveal a substantial individual-specific proteomic response that is not detected to the same degree at the metabolome level (Fig. 1), but also that both complementary nutritional challenges affect a similar set of core proteins.

Five of the plasma proteins, APOC1, APOC2, APOA4, SPP2, and TTR dominated the proteomic nutritional responses to caloric restriction, and APOC1 the response to glucose administration. The 3 apolipoproteins are components of lipoprotein complexes that carry lipids in the circulation, which function to maintain and regulate the assembly, structure, and metabolism of lipoprotein particles (52, 53, 60). APOC1 is part of chylomicrons, very low-density lipoproteins (VLDLs), and HDLs (54), and has been extensively related to lipid metabolism in various ways. Its role in HDL metabolism is to inhibit cholesteryl ester transfer protein (CETP) (55, 56) and activate LCAT (lecithin-cholesterol acyltransferase) (55). An important APOC1 function is the inhibition of lipoprotein lipase (LPL) in the regulation of lipid metabolism (57–62). APOC2 instead binds to the lipoprotein membrane and activates LPL (53, 63), competing with APOC1 and APOC3 and preventing their activity (64).

The proteomic signature was restored quickly upon refeeding in the study participants. Our data is consistent with previous reports (65, 66), which suggest that a single nutritional intervention does not, at least in young, healthy individuals, lead to a long-term signature that is detectable in the plasma proteome. However, our results also show that molecular pathways that have been identified in previous analyses of long-term intervention studies (5, 10) are immediately activated upon a nutritional challenge.

The response to the nutritional challenges further substantiates a connection of metabolism to the innate immune system. A response was recorded for CETP, which regulates the exchange of cholesterol from HDLs with triglycerides of VLDLs (67–69), and the α1-acid glycoprotein ORM2. Serum amyloid P-component (APCS) is an innate immune protein with roles in the regulation of extracellular matrix formation and resistance to infectious agents, and it is an important component of amyloidosis (70). Lumican (LUM), a keratan sulfate proteoglycan, has a role in collagen fibril assembly and connective tissue formation and function (71). Hyaluronan-binding protein 2 (HABP2), a protein that binds to hyaluronan, cleaves the alpha and beta chains of fibrinogen and degrades the extracellular matrix (72), also changes. It also has a protective role in liver fibrosis (73) and was reported in the progression of cancer (74, 75). Individual-specific metabolic and immune responses and correlations with metabolic risk factors can be related to factors such as the gut microbiome (76, 77). Nevertheless, the exploration of the microbiome was not among the aims of this study.

Among all proteins quantified, our plasma proteomic study attributes a particular role to APOC1 as a rapid marker of changes in energy balance. APOC1 was the most rapid responder to glucose ingestion, with its concentration increasing within minutes. Although the Ely cohort individuals is older compared with the CR study, similar nutrient response proteins correlated with the endocrine parameters. The response of APOC1 to glucose consumption is not affected by age in people without diabetes in the Ely study. This is different in individuals with diabetes, where the response is indeed affected by age.

It is plausible that the rapid change in APOC1 results in fast inhibition of LPL, and functions to regulate the formation of triglyceride-rich lipoprotein (Supplementary Fig. S6A (17)). Further, the protein is differentially abundant, and possibly dysregulated in people with diabetes and to some extent in people with prediabetes. This becomes even more evident at recurrent time points. Dysregulation of APOC1 in diabetes was observed also by Bouillet et al (78), although our data indicated a different direction of effect, as we detected APOC1 in lower levels in prediabetes and even lower in people with diabetes, while Bouillet et al (78) found the protein increased. We speculate that these differences could be of technical rather than biological nature: while mass spectrometry can accurately distinguish proteins of high sequence similarity, this is more difficult to achieve by affinity reagents that typically are targeting a single epitope within a protein. Indeed, a decrease in APOC1 could be rationalized by its roles in activating lecithin-cholesterol acyltransferase (LCAT), thus promoting clearance of cholesterol in the liver and inhibiting CETP, a protein targeted in the treatment of type 2 diabetes. CETP regulates the exchange of cholesterol from HDLs with triglycerides of VLDLs (67–69). Its inhibition raises HDL-cholesterol and decreases LDL-cholesterol, which renders it a clinical target (79). Low levels of HDL-cholesterol are associated with insulin resistance (80, 81). Additionally, CETP inhibition resulted in lower glycemic levels (82). The lower levels of APOC1 as detected in diabetes could hence explain a higher CETP activity and be associated with the beneficial effects of CETP inhibitors.

## Conclusions and Limitations of the Study

In summary, we studied the proteomic response to 2 acute nutritional interventions—caloric restriction and an oral glucose tolerance test—as recorded in total 850 human proteomes. We find that the plasma proteome plays a specific part in the acute response to both metabolic interventions, with relatively strong changes in high-abundant plasma proteins. We report that these nutritional interventions have significant individual- and treatment-specific components, but also attribute dominating roles to a converging set of key proteins: APOC1, APOC2, APOA4, SPP2, and TTR. Our data show that the proteins stimulated by the short-term interventions overlap with the those affected by longer-term interventions in previous studies, including weight loss (5, 10), and associate with metabolic parameters in epidemiological studies (Ely and Generation Scotland). Our data put particular emphasis on the role of APOC1. The small, understudied apolipoprotein was affected by both nutritional interventions, in fact dominated the OGTT proteome, and differed in response between individuals without diabetes, with diabetes, and with prediabetes.

To our knowledge, there is a lack of literature about how the human plasma proteome changes with acute nutritional interventions. The novelty of our study also lies in the description of systemic proteomic responses as early as 30, 60, and 120 minutes after a glucose challenge. One limitation of the study is that confounding factors, such as the age of the participants, cannot be easily separated from the effect of nutritional intervention in the Ely study for people with diabetes. Future studies addressing the molecular mechanisms that underlie the proteomic changes seen in this study are needed. Our study reveals the potential of large-cohort proteomics analyses for associating metabolic risk factors and health indicators with specific proteomic profiles and for predicting metabolic responses in health and disease.

## Data Availability

The raw data of the CR (PXD038526, http://www.ebi.ac.uk/pride/archive/projects/PXD038526) (34) and Ely (PXD039023, https://www.ebi.ac.uk/pride/archive/projects/PXD039023) (47) studies will be available via PRIDE (https://www.ebi.ac.uk/pride) (83), a public repository. The processed proteomic of the CR and Ely studies is available in this paper's supplemental information and Mendeley Data repository (17) and will be published upon acceptance. CR metabolomics data are provided by (11) and GS data are suggested by (13). The paper does not report original code.

## Abbreviations

ACN: acetonitrile
APCS: serum amyloid P-component
APO: apolipoprotein
BMI: body mass index
BS: baseline
CETP: cholesteryl ester transfer protein
CR: caloric restriction
DIA: data-independent acquisition
FA: formic acid
FDR: false discovery rate
GS: Generation Scotland study
HDL: high-density lipoprotein
IFG: impaired fasting glucose
IGF2: insulin-like growth factor 2
IGT: impaired glucose tolerance
LDL: low-density lipoprotein
LPL: lipoprotein lipase
MS: mass spectrometry
OGTT: oral glucose tolerance test
PCA: principal component analysis
PLS-DA: partial least square-discriminant analysis
QC: quality control
RF: refeeding
SPP2: secreted phosphoprotein 2
TTR: transthyretin
VLDL: very low-density lipoprotein
